# Chronic kidney disease among overweight and obesity with and without metabolic syndrome in an urban Chinese cohort

**DOI:** 10.1186/s12882-015-0083-8

**Published:** 2015-06-18

**Authors:** Xia Cao, Jiansong Zhou, Hong Yuan, Liuxin Wu, Zhiheng Chen

**Affiliations:** Department of Health Management, the Third Xiangya Hospital, Central South University, Tongzipo Road 138, 410013 Changsha, Hunan Province China; Mental Health Institute, the Second Xiangya Hospital, Central South University, Changsha, Hunan Province China; Department of Clinical Pharmacology Center, the Third Xiangya Hospital, Central South University, Changsha, Hunan Province China; Institute of Aviation Medicine, Beijing, China

**Keywords:** Chronic kidney disease, Metabolic syndrome, Obesity

## Abstract

**Background:**

It is widely accepted that metabolic syndrome is associated with an increased risk of chronic kidney disease (CKD). To investigate whether coexisting metabolic syndrome is a necessary condition for CKD in overweight and obese.

**Methods:**

A cohort study of 6852 Chinese individuals from August 2007 to December 2012. Examinations included a questionnaire, physical measurements, and blood sampling. Hazard ratios for incident CKD were estimated according to combinations of BMI category and absence or presence of metabolic syndrome.

**Results:**

For CKD, multivariable adjusted hazard ratios vs. normal weight individuals without metabolic syndrome were 1.31 (95 % *CI*, 0.89–1.92) in overweight and 2.39 (95 % *CI*, 1.27–4.52) in obese without metabolic syndrome and 1.54 (95 % *CI*, 1.18–3.95) in normal weight, 2.06 (95 % *CI*, 1.27–3.36) in overweight, and 2.77 (95 % *CI*, 1.42–4.31) in obese with metabolic syndrome. There were no interactions between BMI and absence or presence of metabolic syndrome on risk of CKD when BMI was categorized (normal weight, overweight, obese) (*P* = 0.17). Among individuals both with and without metabolic syndrome there were increasing cumulative incidences of CKD from normal weight through overweight to obese individuals (log-rank trend *P* = 0.04 to *P* < 0.001). Although the multivariable adjusted hazard ratio for CKD in individuals with vs. without metabolic syndrome was 1.82 (95 % *CI*, 1.20–2.78) within overweight and obese individuals (log-rank *P* = 0.005), only 26.1 % of the increased risk observed for BMI is explained by metabolic syndrome.

**Conclusions:**

These findings suggest overweight and obesity are risk factors for CKD regardless of the presence or absence of metabolic syndrome.

**Electronic supplementary material:**

The online version of this article (doi:10.1186/s12882-015-0083-8) contains supplementary material, which is available to authorized users.

## Background

The effects of obesity on kidney function and disease have since been identified and become a subject of increased study and concern [1–3]. One plausible mechanism for this link is that overweight and obesity often are accompanied by metabolic syndrome, a cluster of cardiovascular risk factors consisting of hypertension, dyslipidemia, and hyperglycemia [4]. Although there has been considerable disagreement over the diagnostic criteria of metabolic syndrome [5], it is widely accepted that metabolic syndrome is associated with an increased risk of CKD [6–8]. In a large cohort study of >320,000 patients, Hsu *et al.* [9] found that a higher BMI was a strong independent risk factor for end stage renal disease (ESRD) even after adjustment for other major risk factors that are associated with ESRD, including smoking, baseline hypertension, and diabetes. *The question still remains whether coexisting metabolic syndrome is a necessary condition for initiating renal injury in overweight and obese individuals.* Indeed, several studies indicated that obesity may contribute to and perhaps even initiates glomerulopathy [10, 11]. If this is the case, obesity may be as an independent risk factor for the onset, aggravated course, and poor outcomes of CKD, even after adjustment for confounding comorbidities including metabolic syndrome, diabetes and hypertension.

In the present study, we tested the hypothesis that overweight and obesity with and without metabolic syndrome is associated with increased risk of chronic kidney disease. For this purpose, we studied the occurrence of CKD in 6852 subjects without CKD at baseline 2007 through 2012 in central south China and categorized them according to their body mass index (BMI) as normal weight, overweight, or obese and according to absence or presence of metabolic syndrome.

## Methods

### Study cohort

Changsha, the capital city of Hunan province, is a regional hub in central south China. This was a prospective observational study based on a convenient sampling of Chinese urban residents. From August 2007 to December 2012, 10,708 subjects living in this city, between the ages of 20–79 years who underwent their physical check-ups at the Health Management Center of the Third Xiangya Hospital were enrolled in this study. Cohort members in the study were excluded from these analyses if they had a past or present history of CVD at baseline (*n* = 67), were pregnant (*n* = 45), were diagnosed with CKD including each of positive proteinuria (*n* = 612) and estimated glomerular filtration rate (eGFR) < 60 mL/min/1.73 m^2^ (*n* = 1204) at baseline, were missing data (*n* = 1685), or failed to complete the follow-up health surveys or questionnaires after the baseline examination (*n* = 243). After applying these exclusions, a total of 6852 participants aged 20 to 79 years old were selected. The participation rate was 64 %. Examinations included a questionnaire, physical measurements, and blood sampling. All participants provided informed consent before entering the study, and approval was obtained from the Human Subjects Committee at the Third Xiangya Hospital, Central South University.

### Baseline examinations

Seated blood pressures were measured by skilled, trained physicians after subjects had rested for 15 min using a mercury sphygmomanometer according to the American Heart Association’s recommendations [12]. The average of 3 readings was recorded. Blood samples were collected at 08:00–10:00 AM after a fasting period of 12 h. Concentrations of fasting blood glucose (FBG), total cholesterol (TC), and triglycerides were determined by enzymatic colorimetric assay. Highdensity lipoprotein cholesterol (HDL-c) concentration was measured by lipoprotein electrophoresis. Low-density lipoprotein cholesterol (LDL-c) concentration was calculated according to the Friedewald formula. Serum uric acid was determined by an autoanalyzer using the phosphotungstate method. Information on antihypertensive medication, antidiabetic treatment, and lipid-lowering therapy was self-reported. Subjects were identified as current smokers if they had smoked within 1 year of the survey date and physical inactivity was defined as leisure time activity less than 4 h weekly and predominantly sedentary work. BMI was calculated as weight (kg) divided by height squared (m^2^). Individuals were categorized as normal weight with a BMI of 18.5 to 23.9, overweight with BMI of 24.0 to 27.9, and obese with a BMI at least 28.0 according to the Chinese standard [13]. Waist circumference was measured at the midpoint between the lower end of the rib cage and the upper part of the pelvic bone.

### Metabolic syndrome

Metabolic syndrome was defined using the National Cholesterol Education Program Adult Treatment Panel III (NECP-ATP-III) criteria as ≥ 3 of the following 5 metabolic components: 1) elevated waist circumference: ≥ 90 cm (males) or ≥ 80 cm (females); 2) elevated triglycerides: ≥ 1.69 mmol/l or the use of lipid medications; 3) elevated blood pressure: systolic blood pressure ≥ 130 mmHg, or diastolic blood pressure ≥ 85 mmHg, or the use of antihypertensive medications; 4) elevated fasting glucose: ≥ 5.6 mmol/l or the use of diabetes medications; 5) reduced HDL-c: < 1.04 mmol/l (male) or < 1.29 mmol/l (female) [5].

### Definition of CKD

Serum creatinine (Scr) was measured on a Roche/Hitachi Modular System P (Roche Diagnostics GmbH, Mannheim, Germany) by creatinine Jaffe rate blanked and compensated assay. To ensure quality control creatinine was measured in 40 samples with creatinine both at the central laboratory in Hunan province and at the laboratory of Peking University First Hospital. Renal function was estimated by calculated glomerular filtration rate (eGFR) using a modified Chinese equation based on inulin clearance as follows: eGFR (mL/min/1.73 m^2^) = 175 × (Scr in enzymatic method)^-1.234^ × age ^- 0.287^ (×0.79, if female) [14]. Diagnosis of proteinuria was made using a urine dipstick test. Positive proteinuria was considered to be present for a dipstick result of ≥ 1+, corresponding to a urinary protein level > 30 mg/dL [15]. CKD was defined as positive proteinuria or eGFR < 60 mL/min/1.73 m^2^.

### Follow-up and outcomes

Primary outcomes were defined as the onset of CKD at the time of the annual check-up from 2007 to 2012. If a participant experienced more than one CKD event during follow-up, only the first outcome for the individual contributed to the outcome analysis. The date of onset of CKD was defined as the midpoint between the last date when the subjects did not have CKD and the first date when the subject was diagnosed with CKD. The follow-up period was defined as the number of days from the date of observation to the date of CKD diagnosis or to the date of the final check-up. Similarly, an eGFR < 60 mL/min/1.73 m^2^ or an isolated positive proteinuria were analyzed as individual primary outcomes. Secondary outcomes were defined as a composite of CKD or death from all causes.

### Statistical analyses

All statistical analyses were conducted using SAS software, version 8.1 (SAS Institute, Inc.). Analyses of variances and *χ*^2^ tests were used to compare mean values and frequencies. Cumulative incidences were plotted using Kaplan-Meier curves and a log-rank test was used to compare incidence curves. Cox proportional hazards regression models estimated hazard ratios (HRs) for CKD multifactorially adjusted for age, sex, smoking, plasma low-density lipoprotein cholesterol level, antihypertensive/lipid-lowering/antidiabetic medication use, and physical inactivity and the probability value for homogeneity was estimated by adding an interaction term to the statistical model. The percentage of excess risk of CKD for a basic model including BMI and clinical characteristics that was explained by metabolic syndrome was calculated by using the formula: percentage excess risk = [(HR_con adj_ − HR_con + med adj_)/(HR_con adj_ − 1)] × 100 %, where HR_con adj_ is the confounder-adjusted HR for CKD and HR_con + med adj_ is the confounder and mediator–adjusted HR [16].

## Results

Baseline characteristics of the 6852 individuals included in the study are seen in Table 1 stratified by BMI category (normal weight, overweight, obese) and by the absence or presence of metabolic syndrome. Among these, 3864 individuals (56.4 %) were normal weight, 2508 overweight (36.6 %), and 480 individuals (7.0 %) were obese. Metabolic syndrome was present in 6.0 % of normal weight, in 26.2 % of overweight, and in 57.5 % of obese individuals. Characteristics stratified by sex are shown in Tables 2. Among the 3704 male participants, 1432 were normal weight (38.7 %), 1876 were overweight (50.6 %), and 396 individuals (10.7 %) were obese. Corresponding numbers among the 3148 female participants were 2432 (77.3 %), 632 (20.1 %), and 84 (2.7 %), respectively.

**Table 1:** Baseline Characteristics of Participants According to Body Mass Index Categories and Presence or Absence of Metabolic Syndrome

Characteristic	Without Metabolic Syndrome			With Metabolic Syndrome		
	Normal Weight	Overweight	Obese	Normal Weight	Overweight	Obese
Participants, No.	3632	1852	204	232	656	276
Female, %	63.3	23.5	15.7	56.9	29.9	18.8
Age, median (IQR), y	39 (32–47)	47 (37–54)	49 (39–55)	50 (40–60)	49 (42–56)	45 (40–53)
BMI, median (IQR), Kg/m^2^	21.4 (20.0–22.7)	25.4 (24.7–26.4)	29.1 (28.2–29.9)	22.7 (21.2–23.3)	26.2 (25.3–27.1)	29.2 (28.5–30.3)
Waist circumference, median (IQR), cm	75 (68–80)	81 (78–84)	86 (80–92)	80 (78–84)	82 (80–88)	90 (80–96)
Systolic BP, median (IQR), mm Hg	112 (104–122)	122 (114–134)	125 (116–138)	125 (118–138)	130 (122–142)	132 (124–142)
Diastolic BP, median (IQR), mm Hg	69 (62–76)	78 (70–85)	78 (73–88)	80 (71–87)	83 (78–89)	84 (79–90)
Antihypertensive, %	1.9	6.3	15.7	3.4	15.9	21.7
Antidiabetes, %	1.0	2.2	2.8	8.6	3.7	9.8
Lipid-lowering, %	1.4	2.6	5.1	7.8	8.2	10.2
TC, median (IQR), mmol/L	4.4 (3.9–4.9)	4.6 (4.1–5.2)	4.8 (4.2–5.2)	5.1 (4.5–5.8)	4.8 (4.3–5.6)	5.1 (4.4–5.7)
LDL-c, median (IQR), mmol/ L	2.3 (1.9–2.8)	2.6 (2.2–3.1)	2.6 (2.0–3.0)	2.8 (2.3–3.5)	2.5 (1.9–3.2)	2.6 (2.0–3.2)
HDL-c, median (IQR), mmol/L	1.5 (1.3–1.7)	1.3 (1.1–1.5)	1.3 (1.1–1.4)	1.2 (1.0–1.5)	1.1 (1.0–1.3)	1.1 (0.9–1.3)
Triglycerides, median (IQR), mmol/L	0.9 (0.7–1.3)	1.3 (1.0–1.8)	1.5 (1.2–2.2)	1.7 (1.1–2.2)	2.3 (1.8–3.1)	2.4 (1.9–3.0)
Glucose, median (IQR), mmol/L	4.7 (4.4–5.0)	4.8 (4.5–5.2)	4.9 (4.8–5.2)	5.4 (4.7–5.9)	5.3 (4.8–5.8)	5.2 (4.7–6.2)
eGFR, median (IQR), mL/min/1.73 m^2^	104 (82–127)	82 (71–99)	78 (72–93)	90 (76–108)	82 (72–99)	81 (74–90)
Current smokers, %	6.2	29.2	25.5	17.2	23.2	30.0
Physical inactivity, %	39.2	51.0	56.6	46.6	52.4	52.2

Abbreviations: *BMI* body mass index; *IQR*, interquartile range; *BP* blood pressure; *TC* total cholesterol; *LDL-c* Low-density lipoprotein cholesterol; *HDL-c* high-density lipoprotein cholesterol

**Table 2:** Baseline Characteristics of Participants According to Body Mass Index Categories and Sex

Characteristic	Male			Female		
	Normal Weight	Overweight	Obese	Normal Weight	Overweight	Obese
Participants, No.	1432	1876	396	2432	632	84
Age, median (IQR), y	40 (32–53)	48 (38–56)	46 (39–55)	39 (33–45)	47 (39–53)	48 (44–53)
BMI, median (IQR), Kg/m^2^	22.4 (21.0–23.2)	25.7 (24.8–26.7)	29.1 (28.4–30.0)	20.9 (19.7–22.2)	25.4 (24.6–26.4)	29.7 (28.6–31.6)
Waist circumference, median (IQR), cm	78 (76–81)	82 (78–86)	88 (80–94)	71 (66–80)	80 (77–82)	84 (80–87)
Systolic BP, median (IQR), mm Hg	120 (114–130)	126 (118–138)	130 (120–142)	108 (100–118)	120 (114–134)	124 (112–138)
Diastolic BP, median (IQR), mm Hg	74 (66–82)	80 (74–88)	82 (76–90)	68 (60–74)	76 (68–82)	84 (74–88)
Antihypertensive, %	3.4	9.6	18.2	1.1	6.3	23.8
Antidiabetes, %	3.1	3.0	1.0	0.5	1.3	0
Lipid-lowering	3.8	8.1	10.2	1.3	5.2	5.8
TC, median (IQR), mmol/L	4.5 (4.0–5.1)	4.7 (4.2–5.3)	5.0 (4.3–5.4)	4.4 (3.9–4.8)	4.6 (4.1–5.3)	4.9 (4.3–5.2)
LDL-c, median (IQR), mmol/L	2.5 (2.0–3.0)	2.6 (2.1–3.1)	2.6 (2.0–3.0)	2.3 (1.9–2.7)	2.7 (2.1–3.2)	2.5 (2.1–3.0)
HDL-c, median (IQR), mmol/L	1.3 (1.2–1.5)	1.2 (1.0–1.4)	1.1 (1.0–1.3)	1.6 (1.4–1.8)	1.4 (1.2–1.6)	1.4 (1.2–1.7)
Triglycerides, median (IQR), mmol/L	1.2 (0.9–1.8)	1.7 (1.2–2.4)	2.3 (1.6–2.9)	0.9 (0.7–1.1)	1.3 (0.9–1.7)	1.6 (1.2–1.8)
Glucose, median (IQR), mmol/L	4.7 (4.4–5.1)	4.9 (4.6–5.3)	5.1 (4.7–5.6)	4.7 (4.4–5.0)	4.9 (4.5–5.3)	5.1 (4.8–5.4)
eGFR, median (IQR), mL/min/1.73 m^2^	79 (72–91)	77 (70–87)	78 (71–86)	119 (102–139)	114 (98–133)	121 (93–129)
Current smokers, %	36.9	36.9	33.3	0.3	0	0
Physical inactivity, %	46.9	48.5	50.1	38.1	55.1	61.3

Abbreviations: *BMI* body mass index, *IQR* interquartile range, *BP* blood pressure, *TC* total cholesterol, *LDL-c* Low-density lipoprotein cholesterol, *HDL-c* high-density lipoprotein cholesterol

**Fig. 1:**
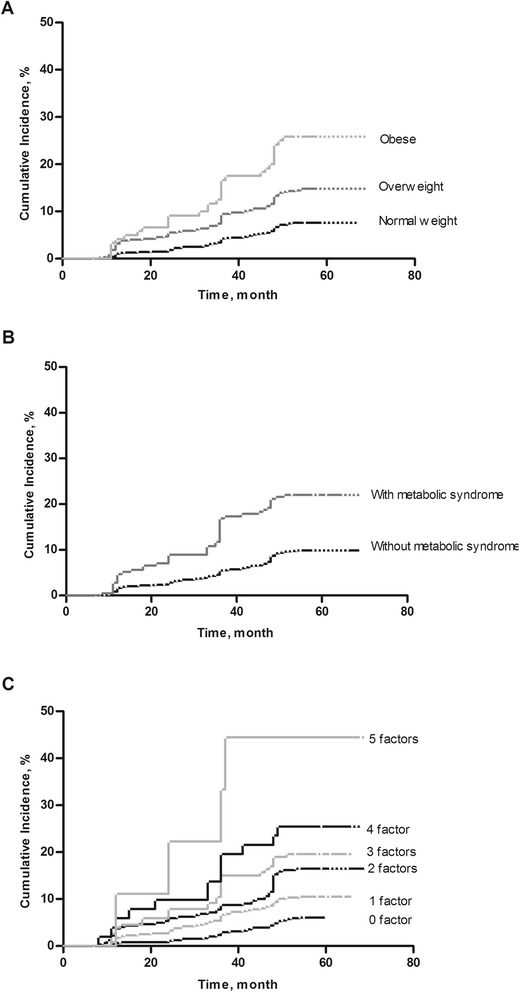
Kaplan-Meier curves for the cumulative incident of chronic kidney disease (CKD) according to body mass index (BMI) category and metabolic syndrome. **a** The hazard ratios (HRs) for chronic kidney disease (CKD) according to body mass index (BMI) category. **b** The HRs for CKD according to metabolic syndrome. **c** The HRs for CKD according to the components of metabolic syndrome

During an average 54.3-month follow-up period, Of the 6852 participants, the number of patients with onset of CKD was 776 subjects, which included solely an eGFR < 60 mL/min/1.73 m2 in 520 (67.0 %) subjects, isolated positive proteinuria in 180 (23.2 %) subjects and both criteria in 76 (9.8 %) subjects. As expected, the cumulative incidences of CKD were higher in overweight and obese vs. normal weight individuals (both log-rank trend *P* < 0.001) (Fig. 1a). Multivariable adjusted HRs for CKD vs. normal weight individuals were 2.04 (95 % CI, 1.50–2.78) in overweight and 3.76 (95 % CI, 2.47–5.71) in obese individuals. Also, the presence of metabolic syndrome was associated with increased cumulative incidences of CKD (log-rank *P* < .001) (Fig. 1b). Multivariable adjusted HRs for CKD in individuals with vs. without metabolic syndrome was 2.42 (95 % CI, 1.74–3.37). Risk of CKD increased stepwise according to the presence of 1, 2, 3, 4, or 5 components of metabolic syndrome (Fig. 1c). The HRs for CKD in individuals vs. with none components of metabolic syndrome were 1.24 (95 % CI, 1.03–1.58), 1.86 (95 % CI, 1.21–2.62), 3.12 (95 % CI, 1.51–6.25), 3.48 (95 % CI, 1.66–7.02), and 5.69 (95 % CI, 2.32–10.31) with one, two, three, four, and five components of metabolic syndrome individuals, respectively. The HRs was adjusted for age, sex, smoking, plasma low-density lipoprotein cholesterol level, antihypertensive/lipid-lowering/antidiabetic medication use, and physical inactivity.

When individuals were divided into groups according to their BMI category (normal weight, overweight, obesity) and absence or presence of metabolic syndrome, risk of CKD increased with higher BMI category independent of presence or absence of metabolic syndrome (Table 3). For CKD, multivariable adjusted HRs vs. normal weight individuals without metabolic syndrome were 1.31 (95 % CI, 0.89–1.92) in overweight and 2.39 (95 % CI, 1.27–4.52) in obese individuals without metabolic syndrome and 1.54 (95 % CI, 1.18–3.95) in normal weight, 2.06 (95 % CI, 1.27–3.36) in overweight, and 2.77 (95 % CI, 1.42–4.31) in obese individuals with metabolic syndrome. There were no interactions between BMI and absence or presence of metabolic syndrome on risk of CKD when BMI was categorized (normal weight, overweight, obese) (*P* = 0.17). Stratifying for sex gave slightly higher risk estimates for CKD in men but attenuated results in women.

**Table 3:** Risk of Chronic Kidney Disease According to Combinations of Body Mass Index (BMI) Category and Absence or Presence of Metabolic Syndrome

BMI Category	Metabolic Syndrome	All			Men			Women		
		Participants, No.	CKD, No.	HR (95 % CI)	Participants, No.	CKD, No.	HR (95 % CI)	Participants, No.	CKD, No.	HR (95 % CI)
Normal weight	No	3632	284	1	1332	185	1	2300	99	1
Overweight	No	1852	248	1.31 (0.89–1.92)	1416	229	1.84 (1.31–2.59)	436	19	1.05 (0.87–1.25)
Obese	No	204	56	2.39 (1.27–4.52)	172	49	3.65 (2.06–5.17)	32	7	1.23 (0.95–1.52)
Normal weight	Yes	232	16	1.54 (1.18–3.95)	100	11	2.19 (1.80–3.66)	132	5	0.88 (0.71–1.27)
Overweight	Yes	656	104	2.06 (1.27–3.36)	460	93	3.13 (1.99–4.90)	196	11	1.35 (1.12–1.64)
Obese	Yes	276	68	2.77 (1.42–4.31)	224	65	4.08 (2.40–5.92)	52	3	1.41 (1.22–1.71)

Hazard ratios were adjusted for age, sex, smoking, plasma low-density lipoprotein cholesterol level, medication use, and physical inactivity. Abbreviations: *95 % CI* 95 % confidence interval, *HR* hazard ratio, *CKD* chronic kidney disease

**Fig. 2:**
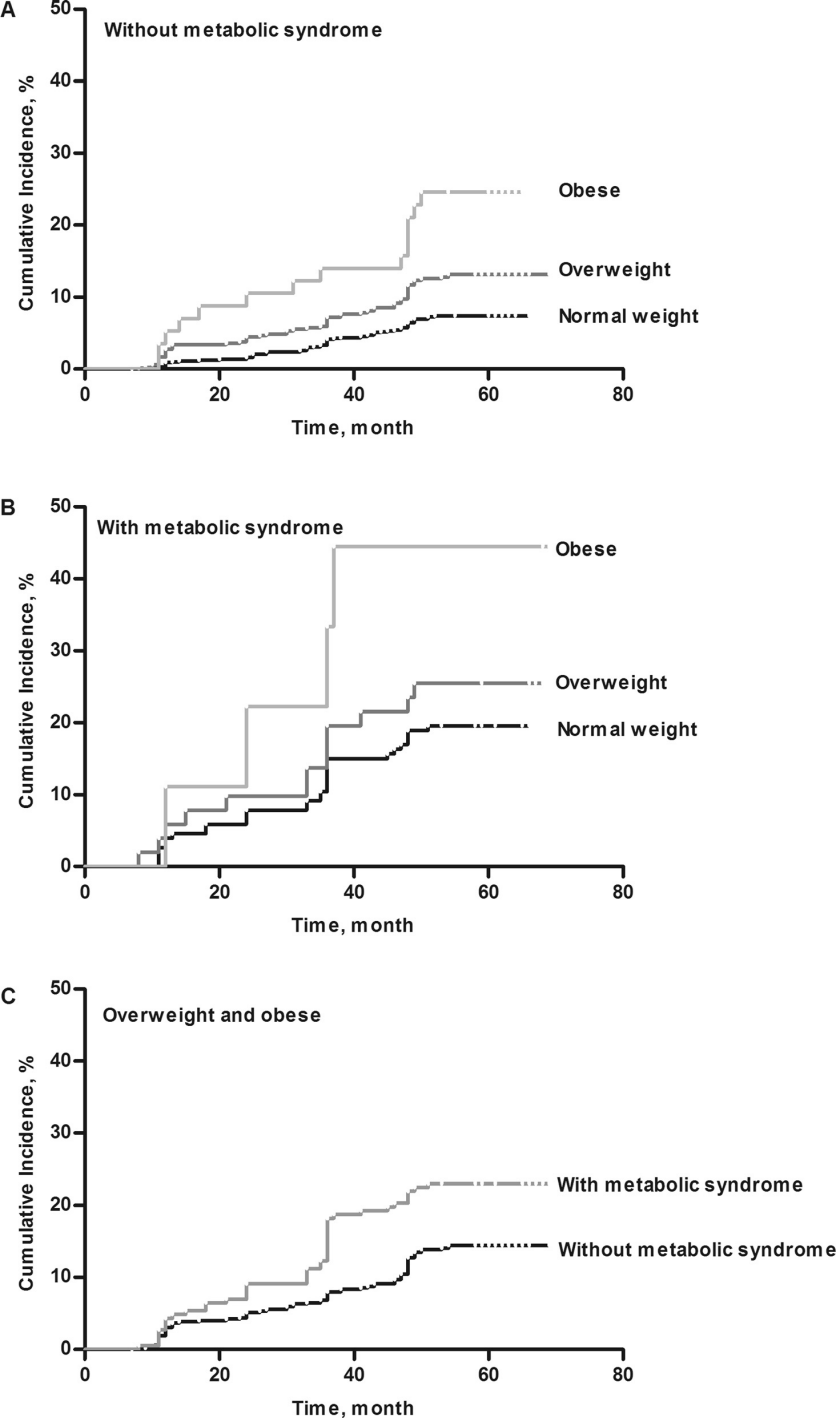
Kaplan-Meier curves for the cumulative incident of chronic kidney disease (CKD) by body mass index category stratified by the absence/presence of the metabolic syndrome. **a** The hazard ratios (HRs) for chronic kidney disease (CKD) by body mass index category without metabolic syndrome. **b** The HRs for CKD by body mass index category with metabolic syndrome. **c** The HRs for CKD with vs. without metabolic syndrome

Among individuals without metabolic syndrome, there were increasing cumulative incidences of CKD from normal weight through overweight to obese individuals (log-rank trend *P* < 0.001) (Fig. 2a). The hazard ratios (HRs) for chronic kidney disease (CKD) vs. normal weight individuals without metabolic syndrome were 1.82 (95 % CI, 1.36–2.42) in overweight and 3.82 (95 % CI, 2.47–6.12) in obese individuals without metabolic syndrome. Among individuals with metabolic syndrome, there likewise were increasing cumulative incidences of CKD from normal weight through overweight to obese individuals (log-rank trend *P* = 0.04) (Fig. 2b). The HRs for CKD vs. normal weight individuals with metabolic syndrome were 1.27 (95 % CI, 1.02–1.68) in overweight and 2.36 (95 % CI, 1.35–3.86) in obese individuals with metabolic syndrome. Within overweight and obese individuals, presence vs. absence of metabolic syndrome was associated with increased cumulative incidences of CKD (log-rank *P* = 0.005) (Fig. 2c). The HRs for CKD with vs. without metabolic syndrome was 1.24 (95 % CI, 1.03–1.58) in overweight and obese individuals.

As shown in Additional file 1: Table S1, the percentage of excess risk mediated by metabolic syndrome in the association between BMI (as a continuous variable) and CKD was 26.1 %; that is, 26.1 % of the associated effect size of BMI on risk of CKD is explained by metabolic syndrome. When BMI was categorized (normal weight, overweight, obese), this proportion increased to 33.3 %.

## Discussion

In this cohort study of a general urban Chinese population, the risk for CKD in overweight and obesity was significantly increased independent of the presence or absence of metabolic syndrome.

Some experimental and clinical evidence have recently implicated a causal link between increased BMI and increased risk of CKD [17, 18]. The suggested mechanism for this link is through obesity-related risk factors such as hypertension, dyslipidemia, and hyperglycemia, all components of metabolic syndrome [19]. Indeed, our findings in the present study may be explained by overweight and obese individuals without metabolic syndrome at baseline developing the other components of the syndrome over time, which later leads to renal injury. In support of this hypothesis, abdominal obesity often precedes the other components [20, 21] and may even be causal in the development of other CKD risk factors such as hypertension [22]. Previous studies have shown that BMI was relatively higher within each BMI category in individuals with metabolic syndrome [23, 24], and this may explain some of the excess risk observed in these individuals compared with those without metabolic syndrome. Also, the addition of metabolic syndrome only slightly increased the capacity of BMI and other clinical characteristics to predict CKD. This was also supported by mediation analyses, which suggested that only a minor proportion of the increased risk observed for BMI is explained by metabolic syndrome. Thus, although the presence of metabolic syndrome increases CKD risk, these associations are not strong and are similar to associations noted between overweight and obesity and incident CKD. This finding has clinical relevance. At subject visits for health care, BMI can easily and accurately be determined with the use of minimum equipment, which would allow the immediate identification of individual at heightened risk that might benefit from therapeutic lifestyle intervention aimed at weight control.

Foster *et al.* [25] found obesity is associated with increased risk of developing stage 3 CKD, which was no longer significant after adjustment for known cardiovascular disease risk factors. In contrast, several studies have found an increased risk of CKD in overweight and obese even after adjustment for baseline covariates [18, 26]. The inconsistencies between these former studies may be caused by differences in defining CKD and/or the cohort selection criteria. Also, studies with small sample sizes or a low number of events may not have enough statistical power to discriminate the differences. To our knowledge, the present study is the largest to date including both men and women in different age groups in Chinese. Our findings suggest that overweight and obesity even in the absence of metabolic syndrome are not benign conditions for renal function.

Obesity may contribute to the development of CKD through pathways related to insulin resistance that can cause renal injury, such as activation of the renin-angiotensin-aldosterone system, activation of insulin/insulin-like growth factor-1 signaling pathways, oxidative stress, suppression of peroxisome proliferatoractivated receptor gamma, inflammation-related tissue damage, nephrosclerosis, and activation of the renal sympathetic system [4, 27]. The glomerular hypertension/hyperfiltration hypothesis as a trigger of CKD progression in overweight/obesity is highlighted by recent observations [28]. Meanwhile, there is mounting evidence that lipids accumulation and alterations in fat cells cytokines can translate into inflammatory changes in the kidney [4].

Some limitations of our study must be considered in evaluating our results. Firstly, our study took place in a single center from a large urban teaching hospital, and the study populations were participants of an annual health check-ups could lead to selection bias due to possible overrepresentation of relatively healthy overweight and obese individuals. Another limitation is that we did not have detail on medication may tend to underestimate the potential effect of agents. Finally, the diagnosis of CKD in the present study depended on the value of creatinine or positive proteinuria on only one occasion and is accordingly more prone to misclassification.

As a global public health problem, obesity is a principal causative factor in the development of metabolic syndrome. However, based on the present data, it is reasonable to suggest that even in the absence of metabolic syndrome, public health strategies should focus on helping individuals to reduce their exposure to an obesity-promoting environment.

Also, because abdominal adiposity seems to precede the development of the other abnormalities in the syndrome, overweight and obesity may in some individuals be an early warning sign for future metabolic disturbances. Animal models have shown that caloric restriction retards kidney injury and these effects may be mediated by up-regulation of Sirt1 expression which is expressed abundantly in the renal inner medulla and in medullary interstitial cells and likely protects medullary interstitial cells from oxidative stress [29, 30]. In addition to this study, in humans with CKD, non-surgical weight loss interventions reduce proteinuria and blood pressure [31]. Moreover, in morbidly obese individuals with glomerular hyperfiltration bariatric surgeries normalize GFR and reduce blood pressure and microalbuminuria [32]. In addition, the antiproteinuric effect of ACE seems to be maximal in overweight/obese patients and less prominent in patients with normal BMI, suggesting role of glomerular hyperfiltration in pathogenesis of proteinuria in obesity [33]. Thus, weight loss and long-term maintenance of that weight loss might be encouraged in overweight and obese individuals, regardless of presence or absence of metabolic syndrome, to reduce risk of obesity-related renal injury.

## Conclusion

In conclusion, overweight and obesity with and without metabolic syndrome are associated with increased risk of CKD in the general population. These findings suggest that overweight and obesity even in the absence of metabolic syndrome are not benign conditions and that weight loss might be encouraged regardless of the presence or absence of metabolic syndrome to reduce the burden of CKD. Our findings also suggest that the presence of metabolic syndrome increases CKD risk, associations are not strong and are similar to associations noted between overweight and obesity and incident CKD however. Although hypertension and diabetes are important mediators, additional pathways also may exist. More clinical trials are needed to assess the impact of overweight and obesity on kidney disease incidence and progression.

## Additional file

Additional file 1: Table S1. Percentage of excess risk for chronic kidney disease mediated by body mass index or the metabolic syndrome.

## Abbreviations

CKD: Chronic kidney disease
BMI: Body mass index
ESRD: End stage renal disease
BMI: Body mass index
eGFR: Estimated glomerular filtration rate
NECP-ATP-III: The National Cholesterol Education Program Adult Treatment Panel III
Scr: Serum creatinin
HRs: Hazard ratios
IQR: Interquartile range
BP: Blood pressure
TC: Total cholesterol
LDL-c: Low-density lipoprotein cholesterol
HDL-c: High-density lipoprotein cholesterol
95 % CI: 95 % confidence interval
