# Supplementary material for: Chronic kidney disease among overweight and obesity with and without metabolic syndrome in an urban Chinese cohort
Source: BMC Nephrol. 2015 Jun 18;16:85. doi: 10.1186/s12882-015-0083-8 (PMC4471928; doi:10.1186/s12882-015-0083-8)
Supplement: Additional file 1: Table S1. — Percentage of excess risk for chronic kidney disease mediated by body mass index or the metabolic syndrome. [file 12882_2015_83_MOESM1_ESM.doc]

Supplemental Table S1. Percentage of excess risk for chronic kidney disease mediated by body mass index or the metabolic syndrome.

| Exposure | Mediator | Hazard ratios (HR) | | Percentage of excess risk mediated, % |
| --- | --- | --- | --- | --- |
|  |  | confounder adjusted | confounder and mediator adjusted |  |
| BMI (continuously) | Metabolic syndrome | 1.03 | 1.02 | 26.1 |
| BMI (categorized) | Metabolic syndrome | 1.51 | 1.34 | 33.3 |
| Metabolic syndrome | BMI (continuously) | 1.67 | 1.56 | 16.4 |
| Metabolic syndrome | BMI (categorized) | 1.67 | 1.38 | 43.3 |

Percentage of excess risk mediated was calculated as ((HR_confounder adjusted_-HR_confounder+mediator adjusted_)/(HR_confounder adjusted_ -1)) × 100 %. Hazard ratios (HRs) were obtained from multivariable adjusted Cox regression analyses. Confounder adjusted models were adjusted for age, sex, smoking, plasma low-density lipoprotein cholesterol, lipid-lowering medication, and physical inactivity. Body mass index (BMI) categorized were as normal weight (18.5-23.9 kg/m^2^), overweight (24–27.9 kg/m^2^), and obese (≥28 kg/m^2^)
